# Supplementary material for: Evolution and modulation of antigen-specific T cell responses in melanoma patients
Source: Nat Commun. 2022 Oct 11;13:5988. doi: 10.1038/s41467-022-33720-z (PMC9553985; doi:10.1038/s41467-022-33720-z)
Supplement: Supplementary file 1 — Supplementary Information [file 41467_2022_33720_MOESM1_ESM.pdf]

Supplementary Fig 1

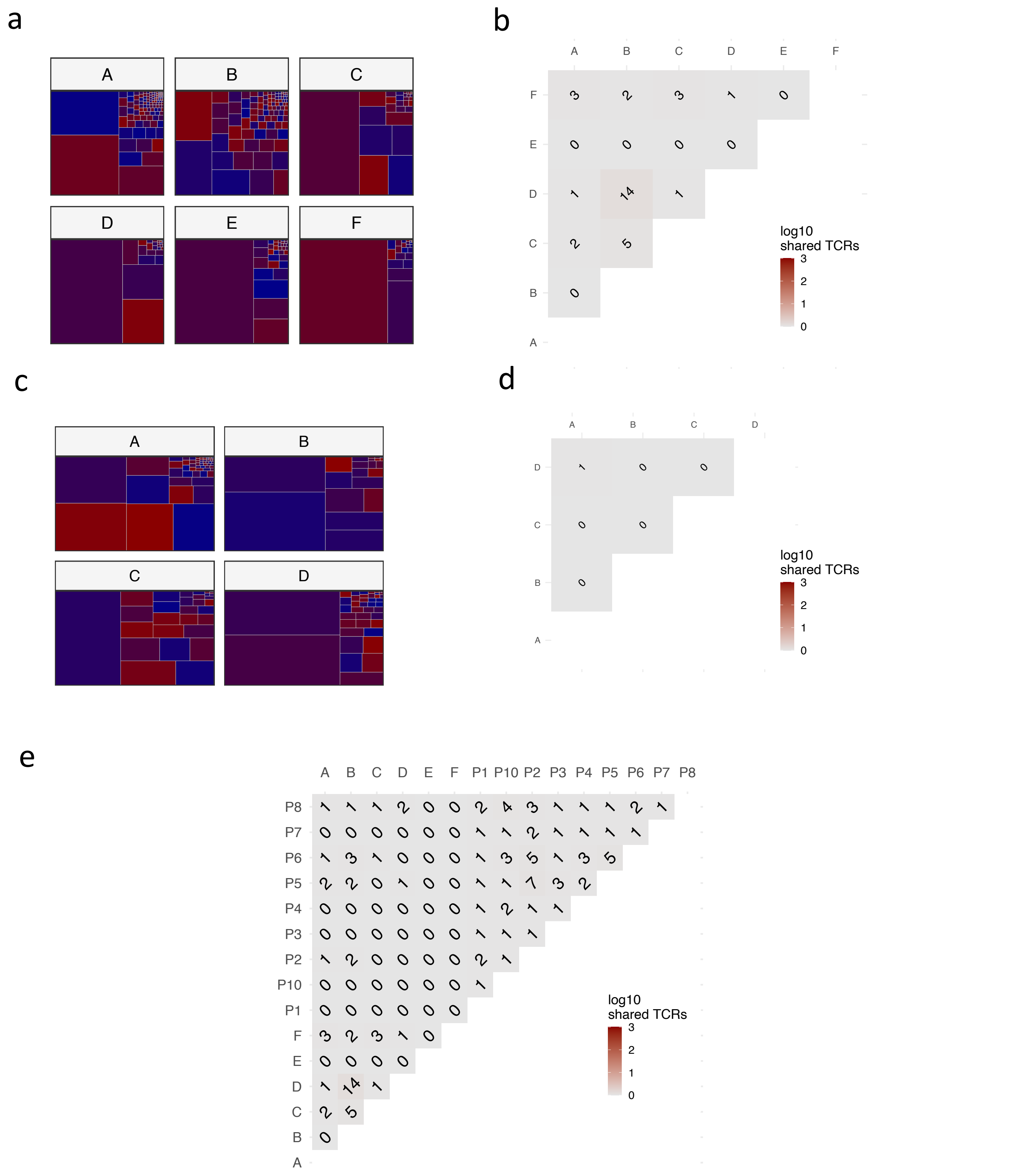

Supplementary Figure 1: Antigen-recognizing signals are conserved between individuals and epitopes and can be learned with machine learning methods

a) Treemap showing the clonotype structure of the HLA-A*02+ restricted MART1$_{ELAGIGILTV}$-repertoire of 6 stage IV melanoma patients enrolled into adoptive cell therapy trial. (Reanalyzed data from[1])
b) Heatmap showing the number of public clonotypes between the MART1$_{ELAGIGILTV}$-infusion products from different patients. Public clonotype was defined as clonotypes that share the same CDR3β amino acid sequences. (Reanalyzed data from[1])
c) Treemap showing the clonotype structure of the HLA-A*02+ restricted MELOE1$_{TLNDECWPA}$-repertoire of 4 stage IV melanoma patients enrolled into adoptive cell therapy trial. (Reanalyzed data from[1])
d) Heatmap showing the number of public clonotypes between the MELOE1$_{TLNDECWPA}$-infusion products from different patients. (Reanalyzed data from[1])
e) Heatmap showing the number of public clonotypes between the infusion products of two similar epitopes, MART1$_{AAGIGILTV}$ and MART1$_{ELAGIGILTV}$. (Reanalyzed data from[1]).

Supplementary Fig 2

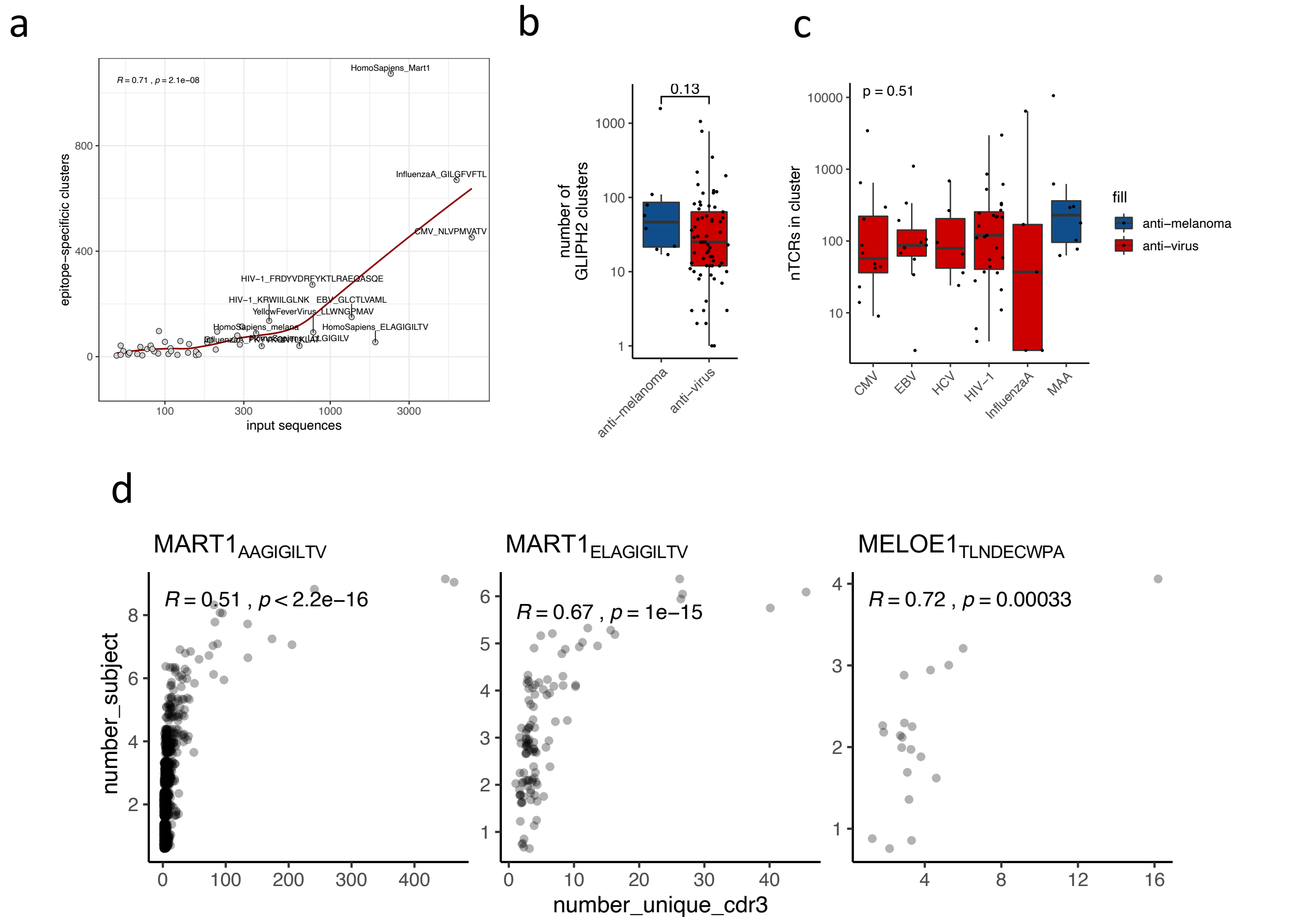

Supplementary Figure 2: GLIPH2 clustering of epitope-specific TCRβ-seq data

a) Scatter plot showing the number of epitope-specific clusters or motifs resolved by GLIPH 2.0 as a function of input sequences. The superimposed line denotes a loess regression model calculated on the data. (Contains data from this study and reanalyzed data from[1,2]).

b) Box plot showing the number of epitope-specific clusters in anti-melanoma (7 epitopes) and anti-viral-specific antigens (70 epitopes). Each dot is one epitope-specific repertoire. *P*-value was calculated with a Mann-Whitney test. (Contains data from this study and reanalyzed data from[1,2]).

c) Box plot showing the number of epitope-specific TCRs in a GLIPH 2.0 predicted cluster in anti-melanoma (7 epitopes) and anti-viral specific antigens (CMV 12 epitopes, EBV 10 epitopes, HCV 6 epitopes, HIV 26 epitopes, Influenza 5 epitopes). Each dot is one epitope-specific repertoire. *P*-value was calculated with a Kruskal-Wallis test. (Contains data from this study and reanalyzed data from[1,2]).

d) Scatter plot showing the publicness of motifs resolved by GLIPH 2.0 for $MART1_{AAGIGILTV}$, $MART1_{ELAGIGILTV}$, and $MELOE1_{TLNDECWPA}$-specific TCRs. Each dot is a motif that is statistically enriched to $MART1_{AAGIGILTV}$-specific TCRβs. The x-axis denotes the number of different CDR3βs that share the given motif, while the y-axis denotes the number of patients that have a CDR3β that is shared between different individuals. *P*-value and correlation coefficient were calculated with a Spearman's rank correlation. (Contains data from this study and reanalyzed data from[1]).

Supplementary Fig 3

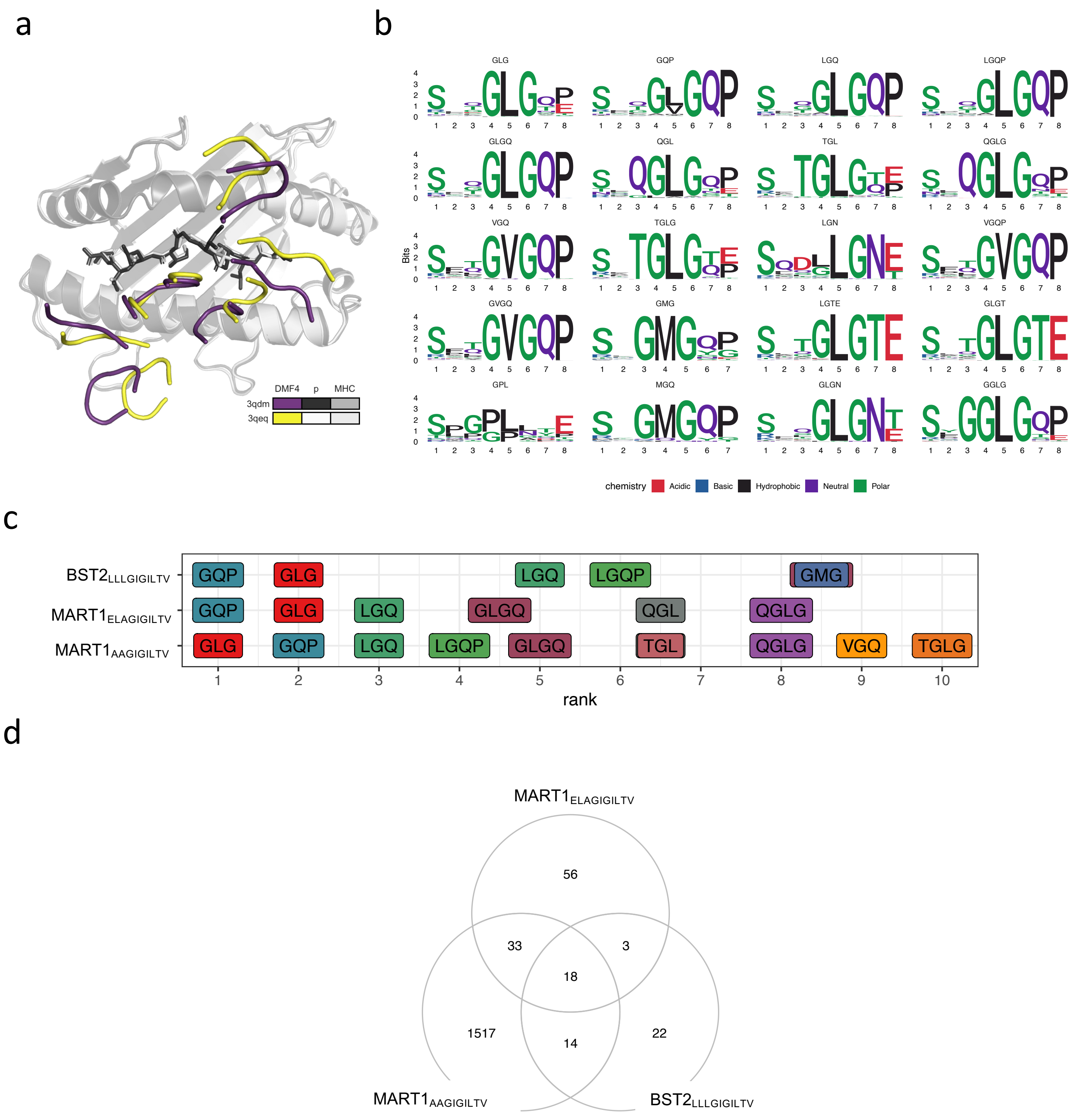

Supplementary Figure 3: Conserved antigen-specific motifs identified by GLIPH2 in similar MAA epitopes

a) Crystallography schematics showing the top view of the superimposition showing the positions of DMF4, a TCR that can bind both MART1$_{ELAGIGILTV}$ (3qdm) and MART1$_{AAGIGILTV}$ (3qdq) epitopes (p) with similar binding. The TCR has the GQP motif, identified by GLIPH2. (Reanalyzed data from[3]).

b) Logo plot showing the amino acid sequences from the antigen-contacting CDR3β of the top 20 motifs measured by the amounts of clustered TCRs in the MART1$_{AAGIGILTV}$-cohort. Letters are colored based on the chemical properties of the amino acids. (Data from this study).

c) Scatter plot showing the GLIPH 2.0 clustering results of the three similar epitopes from two different antigens, MART1$_{AAGIGILTV}$ and MART1$_{ELAGIGILTV}$ epitopes from MART1-antigen and BST2$_{LLLGIGILTV}$ from BST2-antigen as ranks. The ranks are determined by the number of TCRs that share the motif (i.e., rank 1 motif has the most TCR). (Contains data from this study and reanalyzed data from[1,2]).

d) Venn diagram showing the overlap of MART1$_{ELAGIGILTV}$, MART1$_{AAGIGILTV}$, and BST2$_{LLLGIGILTV}$ -specific repertoires. Numbers indicate the numbers of motifs. (Contains data from this study and reanalyzed data from[1,2]).

Supplementary Fig 4

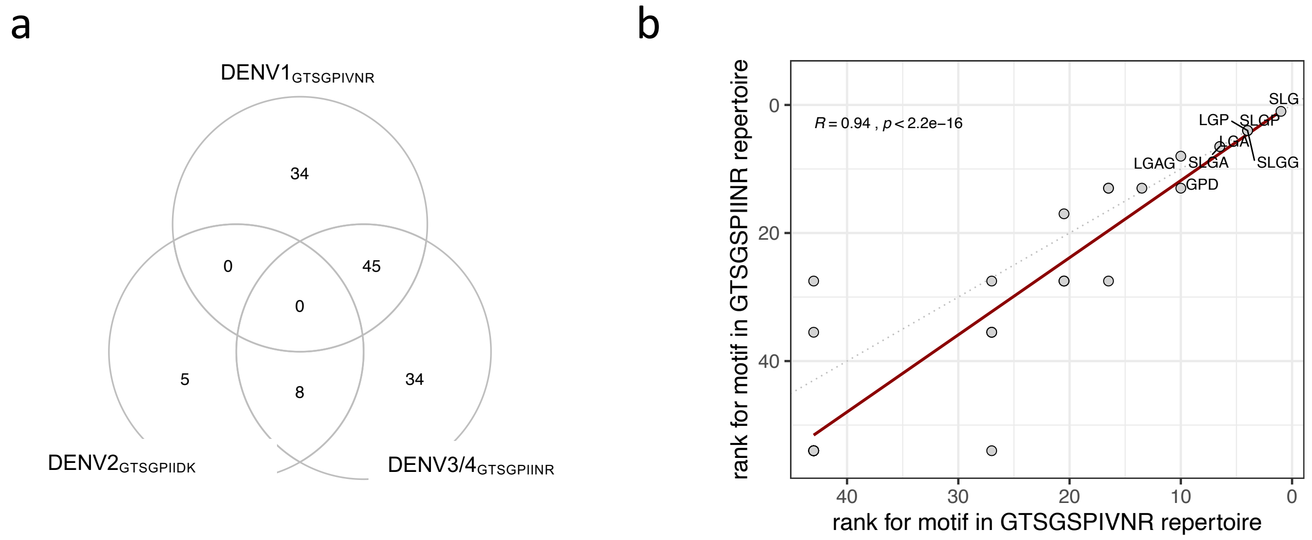

Supplementary Figure 4: Conserved antigen-specific motifs identified by GLIPH2 in similar viral epitopes

a) Venn diagram showing the overlap of similar epitopes from different dengue species, $\mathrm{DENV1}_{\mathrm{GTSGSPIVNR}}$, $\mathrm{DENV2}_{\mathrm{GTSGPIIDK,}}$ and $\mathrm{DENV3/4}_{\mathrm{GTSGPIINR}}$-specific TCRβ-repertoires. Numbers indicate the numbers of motifs. (Reanalyzed data from[2]).
b) Scatter plot showing the motifs resolved by GLIPH 2.0 from the two TCR repertoire of two similar epitopes, $\mathrm{DENV1}_{\mathrm{GTSGSPIVNR}}$, and $\mathrm{DENV3/4}_{\mathrm{GTSGSPIINR}}$. The x-axis denotes the ranks of possible antigen-recognizing motifs from $\mathrm{DENV1}_{\mathrm{GTSGPIVNR}}$-specific TCRs, where the ranks are defined by the number of TCRs that share the motif and the y-axis denotes the same for $\mathrm{DENV3/4}_{\mathrm{GTSGPIINR}}$. Correlation coefficient and *P*-value were calculated with Spearman correlation. (Reanalyzed data from[2]).

Supplementary Fig 5

a

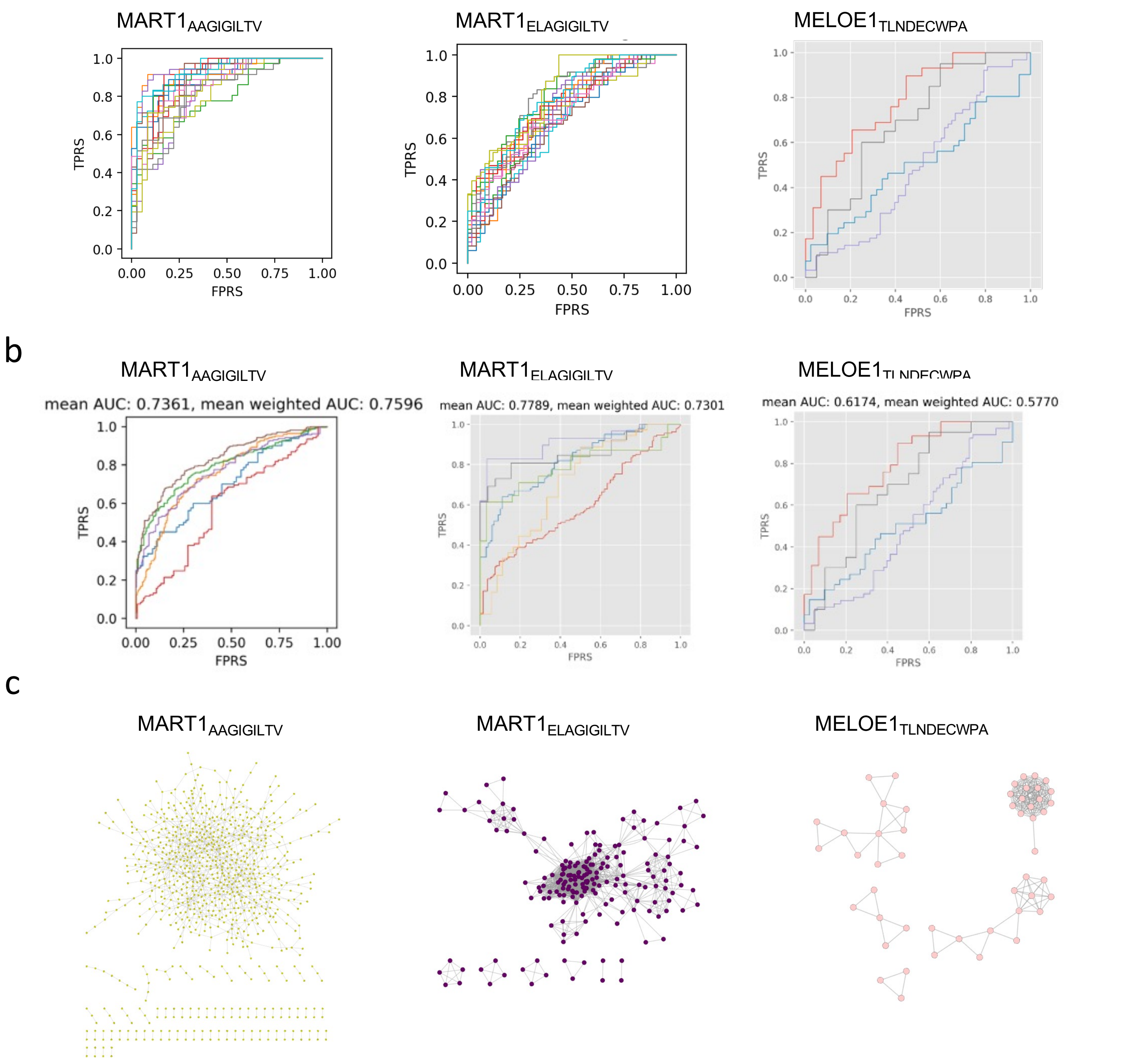

Supplementary Figure 5: Antigen-recognizing signals in anti-MAA clonotypes can be learned with machine learning methods and prediction accuracy correlates with TCR heterogeneity

a) ROC curve plot for $MART1_{AAGIGILTV}$, $MART1_{ELAGIGILTV}$, and $MELOE1_{TLNDECWPA}$-specific TCRBs from the leave-one-fold-out analysis. Each line corresponds to a one left-out fold. (Contains data from this study and reanalyzed data from[1]).

b) ROC curve plot for $MART1_{AAGIGILTV}$, $MART1_{ELAGIGILTV}$, and $MELOE1_{TLNDECWPA}$-specific TCRBs from the leave-one-subject-out analysis. Each line corresponds to a one left-out subject. (Contains data from this study and reanalyzed data from[1]).

c) Network plot showing the GLIPH 2.0 clustering results of pooled $MART1_{AAGIGILTV}$, $MART1_{ELAGIGILTV}$, and $MELOE1_{TLNDECWPA}$-specific TCRβs from all subjects. Each mode is a unique TCRB clonotype, that share identical CDR3β nucleotide sequences as well TCRβV, TCRβD, and TCRβJ gene segments. An edge between nodes denotes a similarity that is statistically enriched to this epitope-specific cluster in comparison to random, non-epitope-specific reference TCRβ repertoire. (Contains reanalyzed data from Simon et al., Front in Immunol 2018). (Contains data from this study and reanalyzed data from[1]).

Supplementary Fig 6

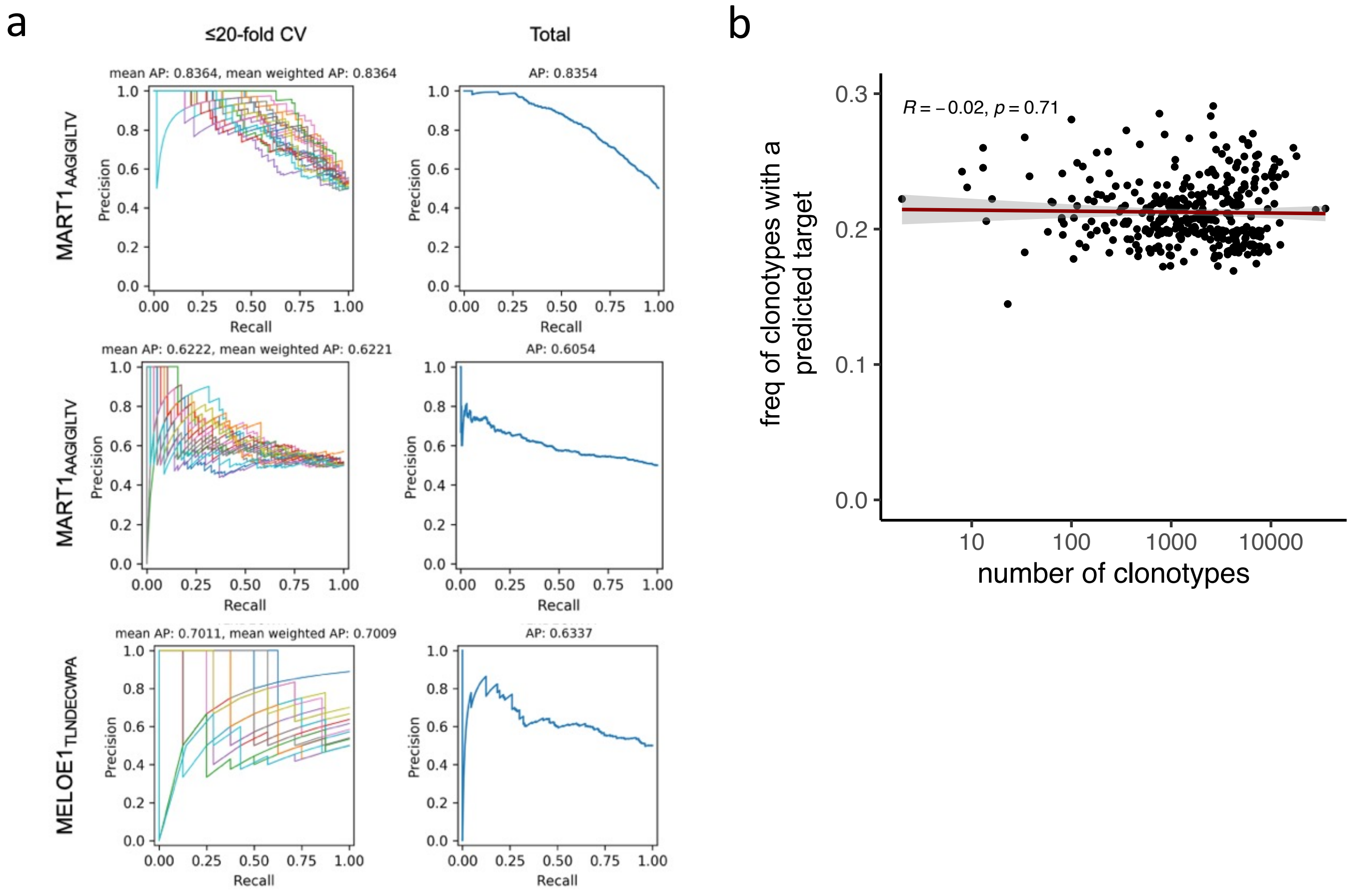

Supplementary Figure 6: The false-positives and false-negatives with the TCRGP classifiers

a) Precision-recall receiver operating characteristic (ROC) curve plots for $MART1_{AAGIGILTV}$, $MART1_{ELAGIGILTV}$, and $MELOE1_{TLNDECWPA}$-specific TCRBs from the leave-one-fold-out analysis (left) and from a total analysis with all data used (right). Each line corresponds to one left-out fold. AP-values correspond to Area under the Precision-Recall Curves. (Contains data from this study and reanalyzed data from[1]).
b) The frequencies of clonotypes with a predicted target against the number of clonotypes in the tumor microenviroment, where each dot is one individual sample (*n*=296). *P*-value and correlation coefficient were calculated with a Spearman's rank correlation. (Contains data from this study and reanalyzed data from[4–6]).

Supplementary Fig 7

a

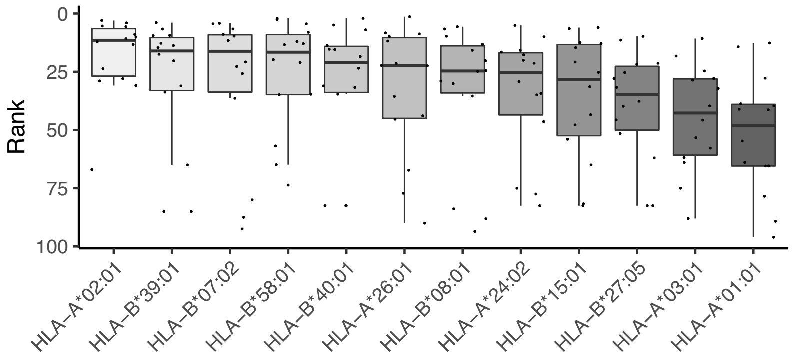

Supplementary Figure 7: Prediction of epitopes with ***GIGILTV binding to different HLA supertypes

a) The NetMHCpan-4.0 rank results for binding of epitopes containing ***GIGILTV motif, which is the stretch of amino acids shared between the $MART1_{AAAGIGILTV}$ and $MART1_{ELAGIGILTV}$ epitopes, by different HLA supertypes.

Supplementary Fig 8

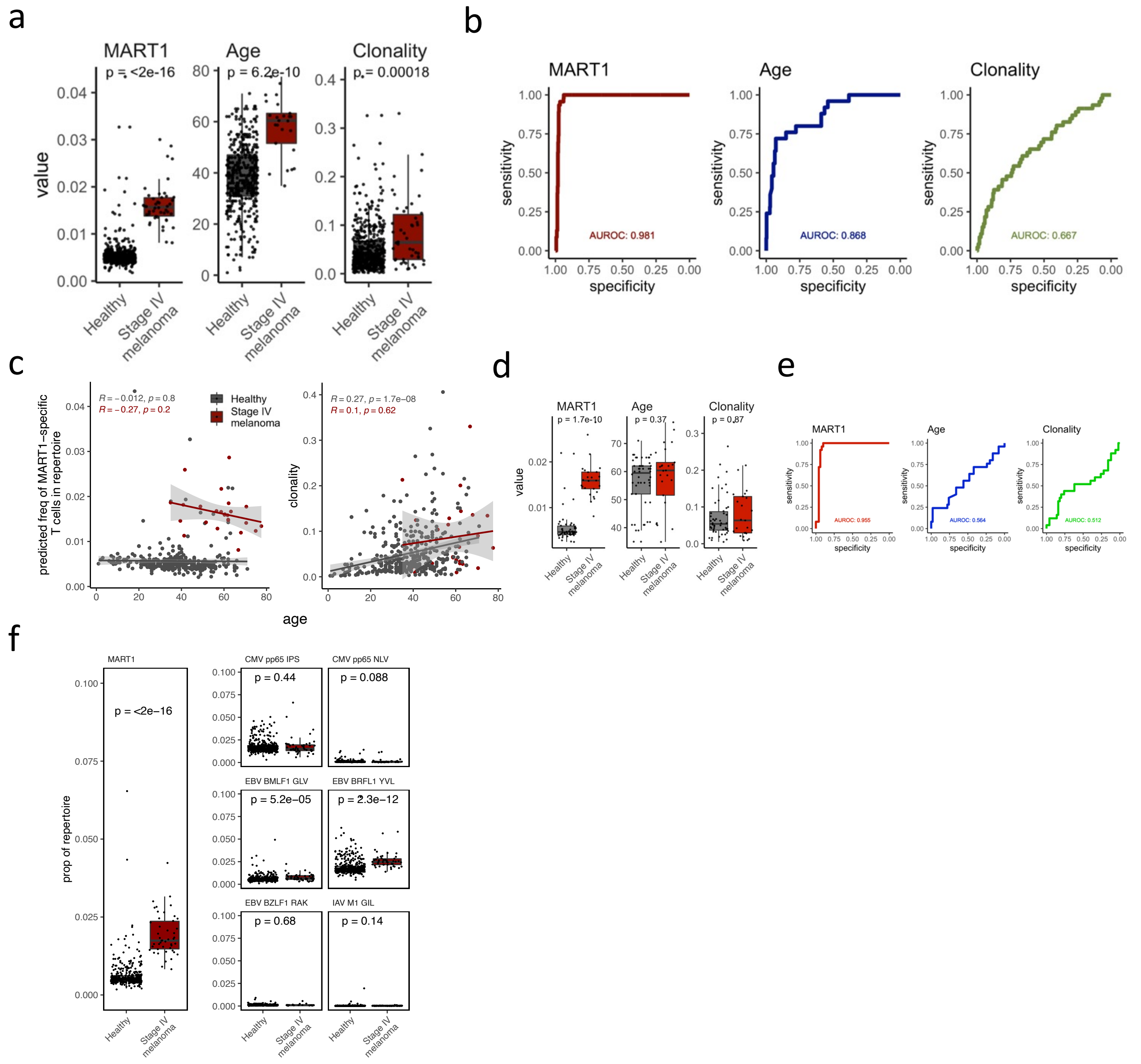

Supplementary Figure 8: Anti-MAA TCRs can be used to separate patients with melanoma from healthy from a blood sample

a) The proportion of predicted MART1-specific clonotypes (left), age (middle), and clonality (right) from healthy donors (*n*=783) and stage IV melanoma patients (*n*=46) sampled from peripheral blood. *P*-value was calculated with two-sided Mann-Whitney test. (Contains data from this study and reanalyzed data from[6–8]).
b) Receiver operating characteristic (ROC) curve plots showing the ROC curves for separating stage IV melanoma patients from healthy from peripheral blood samples by using the the proportion of predicted MART1-specific clonotypes (left), age (middle), or TCR repertoire clonality (right). (Contains data from this study and reanalyzed data from[6–8]).
c) The correlation between donor age and the proportion of predicted MART1-specific clonotypes (left) and the TCR repertoire clonality (right) from healthy donors (*n*=783) and stage IV melanoma patients (*n*=46) sampled from peripheral blood. *P*-value and correlation coefficient were calculated with a Spearman's rank correlation. (Contains data from this study and reanalyzed data from[6–8]).
d) Same as in panel A but for age-matched cohort (stage IV melanoma patients *n*=25, healthy donors *n*=50).
e) Same as in panel B but for age-matched cohort (stage IV melanoma patients *n*=25, healthy donors *n*=50).
f) The abundances of predicted MART1-specific clonotypes and CMV pp65$_{IPSINVHHY}$, CMV pp65$_{NLVPMVATV}$, EBV BMLF1$_{GLCTLVAML}$, EBV BRFL1$_{YVLDHLIVV}$, EBV BZLF1$_{RAKFKQLL}$, Influenza A M1$_{GILGFVFTL}$ from healthy donors (*n*=783) and stage IV melanoma patients (*n*=46) sampled from peripheral blood. *P*-value was calculated with two-sided Mann-Whitney test. (Contains data from this study and reanalyzed data from[6–8]).

Supplementary Fig 9

a

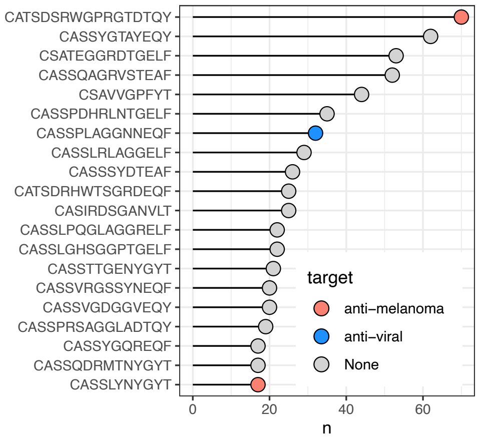

b

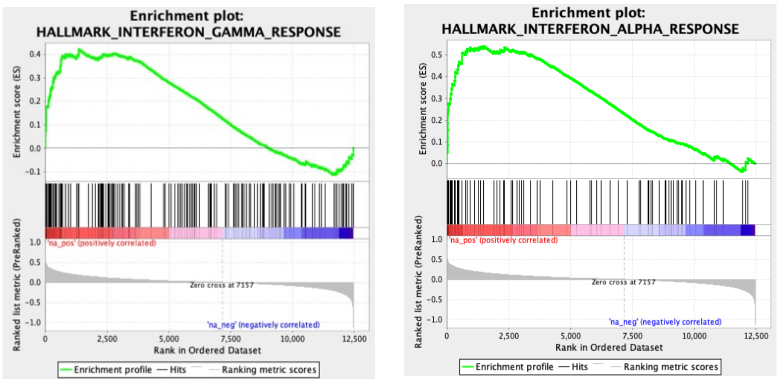

Supplementary Figure 9: The largest clonotype in the Li et al data was predicted to be MAA-specific and MAA-specific cells upregulated IFN-γ and IFN-α pathways

a) The 20 expanded clonotypes in the Li et al. dataset and their TCRGP predicted antigen-specificities. (Reanalysed data from[9]).
b) The enrichment plots of statistically significant IFNγ and IFNα response -pathways in TCRGP-predicted anti-MAA clonotypes in comparison to anti-viral clonotypes. (Reanalysed data from[9]).

Supplementary Fig 10

a

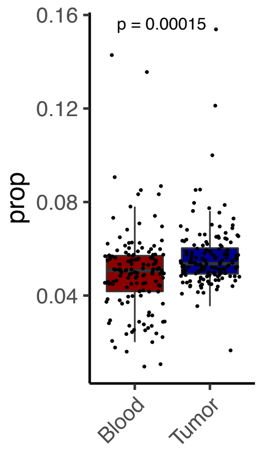

Supplementary Figure 10: The proportion of anti-MAA T cells in blood and tumor from patients with melanoma

a) The proportion of predicted anti-MAA clonotypes from patients with melanoma sampled from blood (*n*=113) or from tumor *(n*=113*). P*-value was calculated with a two-sided Mann-Whitney test. (Contains data from this study and reanalyzed data from[4–7]).

Supplementary Fig 11

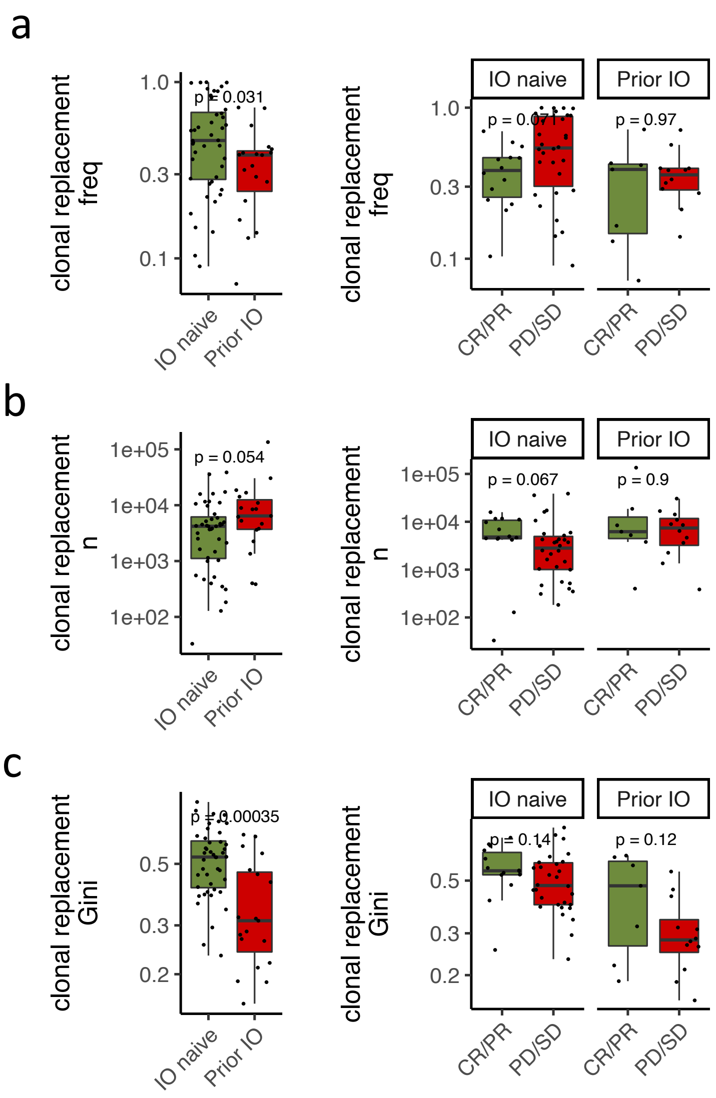

Supplementary Figure 11: Clonal replacement is not associated with response to immune checkpoint therapy in melanoma

a) The frequency, **b)** number, and **c)** clonality of replaced clones following immune checkpoint therapy in the tumor microenvironment in melanoma patients. Samples are separated whether the patients were treated with immune checkpoint therapy as a first-line therapy (IO naïve, *n*=46) or patients previously treated unsuccessfully with immunotherapy (Prior IO, *n=15*) (left; or response status (right; IO naïve CR/PR *n*=18, SD/PD *n*=28; prior IO CR/PR *n*=4, SD/PD *n*=10). (Contains data from this study and reanalyzed data from[4–7]).

Supplementary Fig 12

a

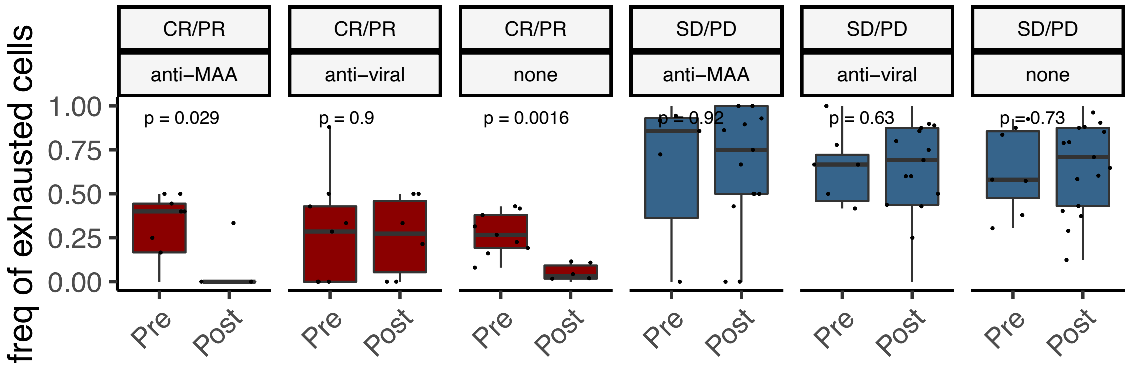

Supplementary Figure 12: Immune checkpoint-therapies reverse the exhaustion anti-MAA clonotypes in responders

a) Boxplot showing the frequency of exhausted cells before and after immune-checkpoint therapy. Patients were divided by the response (responders *n*=18, including complete response [CR] and partial response [PR], non-responders *n*=14, including stable disease [SD], and progressive disease [PD], defined by RECIST criteria). T cells were divided by TCRGP predicted specificities, where melanoma-specific clonotypes include T cells that were predicted by their CDR3β part to be reactive against epitopes from melanoma-associated antigens MART1$_{AAGIGILTV,}$ MART1$_{ELAGIGILTV,}$ MELOE1$_{TLNDECWPA}$, TKT$_{AMFWSVPTV,}$ and SEC24A$_{FLYNLLTRV}$. Similarly, the viral-specific antigens include CMV pp65$_{IPSINVHHY}$, CMV pp65$_{NLVPMVATV}$, CMV pp65$_{TPRVTGGGAM}$, EBV BMLF1$_{GLCTLVAML}$, EBV BRLF1$_{YVLDHLIVV,}$ EBV BZLF1$_{RAKFKQLL,}$ and InfA M1$_{GILGFVFTL}$. *P*-values were calculated with paired Mann-Whitney test. (Reanalyzed data from[10]).

References

1. Simon, S. *et al.* TCR Analyses of Two Vast and Shared Melanoma Antigen-Specific T Cell Repertoires: Common and Specific Features. *Front. Immunol.* **9**, 1962 (2018).

2. Shugay, M. *et al.* VDJdb: a curated database of T-cell receptor sequences with known antigen specificity. *Nucleic Acids Res.* **46**, D419–D427 (2018).

3. Borbulevych, O. Y., Santhanagopolan, S. M., Hossain, M. & Baker, B. M. TCRs used in cancer gene therapy cross-react with MART-1/Melan-A tumor antigens via distinct mechanisms. *J. Immunol.* **187**, 2453–2463 (2011).

4. Tumeh, P. C. *et al.* PD-1 blockade induces responses by inhibiting adaptive immune resistance. *Nature* **515**, 568–571 (2014).

5. Riaz, N. *et al.* Tumor and Microenvironment Evolution during Immunotherapy with Nivolumab. *Cell* **171**, 934-949.e16 (2017).

6. Yusko, E. *et al.* Association of Tumor Microenvironment T-cell Repertoire and Mutational Load with Clinical Outcome after Sequential Checkpoint Blockade in Melanoma. *Cancer Immunol Res* **7**, 458–465 (2019).

7. Robert, L. *et al.* CTLA4 blockade broadens the peripheral T-cell receptor repertoire. *Clin. Cancer Res.* **20**, 2424–2432 (2014).

8. Emerson, R. O. *et al.* Immunosequencing identifies signatures of cytomegalovirus exposure history and HLA-mediated effects on the T cell repertoire. *Nat. Genet.* **49**, 659–665 (2017).

9. Li, H. *et al.* Dysfunctional CD8 T cells form a proliferative, dynamically regulated compartment within human melanoma. *Cell* **181**, 747 (2020).

10. Sade-Feldman, M. *et al.* Defining T Cell States Associated with Response to Checkpoint Immunotherapy in Melanoma. *Cell* **175**, 998-1013.e20 (2018).
