# Supplementary material for: Evolution and modulation of antigen-specific T cell responses in melanoma patients
Source: Nat Commun. 2022 Oct 11;13:5988. doi: 10.1038/s41467-022-33720-z (PMC9553985; doi:10.1038/s41467-022-33720-z)
Supplement: Supplementary file 3 — Description of Additional Supplementary Files [file 41467_2022_33720_MOESM3_ESM.pdf]

Supplementary Data 1: The data sets used in the project and the clinical data related to anti-LAG3+anti-PD1 trial

Supplementary Data 2: The correlation analysis of shared TCR motifs across epitope-specific TCR repertoires.

Supplementary Data 3: The differentially expresed genes between clusters, anti-MAA and anti-viral T cells, and genes differentially expressed along pseudotime

Supplementary Data 4: The statistically significant ligand-receptor interactions

Supplementary Data 5: The multivariate and univariate analysis of overall survival in primary melanoma

Supplementary Data 6: The expanded TCR clonotypes and their targets
