# Supplementary material for: Evolution and modulation of antigen-specific T cell responses in melanoma patients
Source: Nat Commun. 2022 Oct 11;13:5988. doi: 10.1038/s41467-022-33720-z (PMC9553985; doi:10.1038/s41467-022-33720-z)
Supplement: Supplementary file 10 — Reporting Summary [file 41467_2022_33720_MOESM10_ESM.pdf]

nature portfolio

Corresponding author(s): Satu Mustjoki; Mark M. Davis

Last updated by author(s): Feb 9, 2022

Reporting Summary

Nature Portfolio wishes to improve the reproducibility of the work that we publish. This form provides structure for consistency and transparency in reporting. For further information on Nature Portfolio policies, see our Editorial Policies and the Editorial Policy Checklist.

Statistics

For all statistical analyses, confirm that the following items are present in the figure legend, table legend, main text, or Methods section.

n/a	Confirmed	
☐	☒	The exact sample size (n) for each experimental group/condition, given as a discrete number and unit of measurement
☐	☒	A statement on whether measurements were taken from distinct samples or whether the same sample was measured repeatedly
☐	☒	The statistical test(s) used AND whether they are one- or two-sided<br>*Only common tests should be described solely by name; describe more complex techniques in the Methods section.*
☐	☒	A description of all covariates tested
☐	☒	A description of any assumptions or corrections, such as tests of normality and adjustment for multiple comparisons
☒	☐	A full description of the statistical parameters including central tendency (e.g. means) or other basic estimates (e.g. regression coefficient) AND variation (e.g. standard deviation) or associated estimates of uncertainty (e.g. confidence intervals)
☐	☒	For null hypothesis testing, the test statistic (e.g. F, t, r) with confidence intervals, effect sizes, degrees of freedom and P value noted<br>*Give P values as exact values whenever suitable.*
☒	☐	For Bayesian analysis, information on the choice of priors and Markov chain Monte Carlo settings
☒	☐	For hierarchical and complex designs, identification of the appropriate level for tests and full reporting of outcomes
☒	☐	Estimates of effect sizes (e.g. Cohen's d, Pearson's r), indicating how they were calculated

Our web collection on statistics for biologists contains articles on many of the points above.

Software and code

Policy information about availability of computer code

Data collection	No software was used for data collection.
Data analysis	R (4.0.2) or Python (3.7.4), Cell Ranger (ver 2.1.1), scVI (ver 0.5.0), SingleR (ver 1.2.4), Seurat (ver 3.0.0), ClusterProfiler (3.16.0), CellPhoneDB (ver 2.0.0), VDJtools (ver 1.2.1), GLIPH2 (1.0.0), TCRGP (ver 1.0.0), finalfit (v 1.0.5), were used for data analysis. The code to reproduce the key findings is available in https://github.com/janihuuh/melanomap_manu

For manuscripts utilizing custom algorithms or software that are central to the research but not yet described in published literature, software must be made available to editors and reviewers. We strongly encourage code deposition in a community repository (e.g. GitHub). See the Nature Portfolio guidelines for submitting code & software for further information.

Data

Policy information about availability of data

All manuscripts must include a data availability statement. This statement should provide the following information, where applicable:

- Accession codes, unique identifiers, or web links for publicly available datasets
- A description of any restrictions on data availability
- For clinical datasets or third party data, please ensure that the statement adheres to our policy

The TCRβ-sequencing data and Seurat-objects are available at Zenodo under DOI: 10.5281/zenodo.6882576 with restricted access due to General Data Protection Regulation (GDPR) regulations and data can be accessed by placing a request via Zenodo to the leading and corresponding authors and will be reviewed without undue delay. Additionally, the TCRB-sequencing data are available at immuneAccess under DOI: 10.21417/JH2022NC (http://clients.adaptivebiotech.com/pub/huuhtanen-2022-nc). The publicly available scRNA+TCRαβ-sequencing and TCRβ-sequencing data used in this study are listed in Supplementary Data 1. Source data are provided with this paper. The remaining data are available within the Article, Supplementary Information or Source Data file.

Field-specific reporting

Please select the one below that is the best fit for your research. If you are not sure, read the appropriate sections before making your selection.

[x] Life sciences   [ ] Behavioural & social sciences   [ ] Ecological, evolutionary & environmental sciences

For a reference copy of the document with all sections, see nature.com/documents/nr-reporting-summary-flat.pdf

Life sciences study design

All studies must disclose on these points even when the disclosure is negative.

Sample size	Sample size calculations were not performed. We collected scRNA+TCRab-seq, bulk-RNA-seq and TCRb-seq from available published melanoma at the time of study initiation.
Data exclusions	No data was excluded.
Replication	For different analyses, findings were replicated in a validation cohort when available. Especially findings related to MART1AAGIGILTV-specific reperotire, findings werereplicated with 75 different epitopes. For findings related to cutaneous melanoma scRNA-seq data, the findings were replicated with a cutaneous melanoma scRNA-seq data and uveal melanoma scRNA-seq data. For TCRGP model building, separate training and test sets were separated as stated in the methods. To assess the models, we used either leave-one-out cross-validation with epitopes with <100 epitope-specific TCRs and 20-fold cross-validation for epitopes with more TCRs (Supplementary Data 1). The anti-MAA classifiers were also assessed in leave-one-subject-out cross-validation scheme (Supplementary Figure 5b) and as precision-recall AUROCs with 1:1 of positive and negative samples, from which we have also included the Area under the Precision-Recall Curves (AP-values54) as summary values (Supplementary Figure 6a).
Randomization	No randomization was performed, as controlling for covariates was not possible as they were not available for all data sets and no intervention for the study participants was offered by the authors.
Blinding	No blinding was performed as they are not commonly performed on preclinical studies due to

Reporting for specific materials, systems and methods

We require information from authors about some types of materials, experimental systems and methods used in many studies. Here, indicate whether each material, system or method listed is relevant to your study. If you are not sure if a list item applies to your research, read the appropriate section before selecting a response.

Materials & experimental systems

n/a	Involved in the study	
[x]	[ ]	Antibodies
[x]	[ ]	Eukaryotic cell lines
[x]	[ ]	Palaeontology and archaeology
[x]	[ ]	Animals and other organisms
[ ]	[x]	Human research participants
[ ]	[x]	Clinical data
[x]	[ ]	Dual use research of concern

Methods

n/a	Involved in the study	
[x]	[ ]	ChIP-seq
[x]	[ ]	Flow cytometry
[x]	[ ]	MRI-based neuroimaging

Human research participants

Policy information about [studies involving human research participants]()

Population characteristics	This study included 33 metastatic melanoma patients who were treated at the Helsinki University Hospital Comprehensive Cancer Center, Finland. All other participants had been recruited previously, and the population details can be found in Supplementary Data 1, including prior treatment, age, and CMV seropositivity.
Recruitment	The patients were recruited as a part of an ongoing unpublished phase I study, where recruitment criteria can be seen in the original trial protocol available at NCT01968109 The study was approved by Helsinki University Central Hospital (HUCH) ethical committee (Dnro 115/13/03/02/15). Written informed consent was received from all patients and the study was conducted in accordance with the Declaration of Helsinki.
Ethics oversight	The study was approved by Helsinki University Central Hospital (HUCH) ethical committee (Dnro 115/13/03/02/15).

Note that full information on the approval of the study protocol must also be provided in the manuscript.

Clinical data

Policy information about clinical studies

All manuscripts should comply with the ICMJE guidelines for publication of clinical research and a completed CONSORT checklist must be included with all submissions.

Clinical trial registration	NA
Study protocol	NA
Data collection	NA
Outcomes	NA
